# Modified Percutaneous Endoscopic Interlaminar Discectomy through the Near‐spinous Process Approach for L4/5 Disc Herniation: A Retrospective Clinical Study

**DOI:** 10.1111/os.14031

**Published:** 2024-03-31

**Authors:** Peichuan Xu, Jinghong Yuan, Tianlong Wu, Dingwen He, Xinxin Miao, Xigao Cheng

**Affiliations:** ^1^ Department of Orthopedics The Second Affiliated Hospital of Nanchang University Nanchang China; ^2^ Institute of Orthopedics of Jiangxi Province Nanchang China

**Keywords:** L4/5 Lumbar disc herniation, Percutaneous endoscopic interlaminar discectomy, Percutaneous endoscopic lumbar discectomy, Percutaneous endoscopic transforaminal discectomy

## Abstract

**Objective:**

Compared with traditional open surgery, percutaneous endoscopic lumbar discectomy (PELD) has the advantages of less trauma, faster recovery, and less postoperative pain, so it has been widely used in the field of spinal surgery. However, it still has the defect of intraoperative fluoroscopy occurrences, complications, and even the risk of damage to the spinal cord and nerve. This study aims to compare the clinical efficacy of modified percutaneous endoscopic interlaminar discectomy (MPEID) with percutaneous endoscopic transforaminal discectomy (PETD) in treating L4/5 lumbar disc herniation (LDH) and to evaluate the effectiveness and safety of MPEID.

**Methods:**

Thirty‐four L4/5 LDH patients treated at the Second Affiliated Hospital of Nanchang University from June 2020 to June 2021 were studied retrospectively. Seventeen underwent MPEID and seventeen PETD. Variables analyzed included demographics, operative duration, intraoperative fluoroscopy occurrences, and surgical outcomes. Effectiveness was evaluated using the visual analogue scale (VAS), Oswestry disability index (ODI), and modified MacNab criteria. Lumbar Magnetic Resonance Imaging (MRI) was used to assess radiological outcomes. A paired *t*‐test was performed to compare intragroup pre‐ and postoperative clinical data, VAS, and ODI scores.

**Results:**

The average operative time in PETD group was 91.65 ± 14.04 min, and the average operative time in MPEID group was 65.41 ± 12.61 min (*p* < 0.001). In PETD group, the fluoroscopy occurrences averaged 9.71 ± 1.05 times, with fluoroscopy occurrences averaging 6.47 ± 1.00 times (*p* < 0.001) in MPEID group. At 12 months follow‐up, the clinical effect showed significant improvement in both two groups. The MPEID group showed a decrease in average VAS‐back score from 5.41 ± 2.18 to 1.76 ± 1.09 (*p* < 0.001) and VAS‐leg score from 6.53 ± 1.66 to 0.82 ± 0.64 (*p* < 0.001). The ODI scores decreased from 51.35 ± 10.65 to 11.71 ± 2.91 (*p* < 0.001). In the PETD group, the VAS‐back score decreased from 4.94 ± 1.98 to 2.06 ± 1.25 (*p* < 0.001), VAS‐leg score from 7.12 ± 1.73 to 1.12 ± 0.60 (*p* < 0.001), and ODI scores from 48.00 ± 11.62 to 12.24 ± 2.56 (*p* < 0.001). According to the modified MacNab criteria, MPEID had 15 excellent and two good results; PETD had 12 excellent and 5 good (*p = 0.23*). No nerve root injuries, dural tears, or significant complications were reported.

**Conclusion:**

MPEID and PETD effectively treat L4/5 LDH, with MPEID showing shorter operative times and fewer fluoroscopies. Furthermore, the MPEID group can provide excellent clinical efficacy as the PETD group in the short term.

## Graphical abstract

Schematic diagram of the modified percutaneous endoscopic interlaminar discectomy (MPEID) spinal endoscopy technique for L4/5 disc herniation. (A) and (B) Interlaminar space with and without a postural cushion on X‐ray. (C) An 18‐G needle was inserted from the lower end of the L5 spinous process to the most lateral edge of the L4 lamina on the affected side. (D) The sequential tract dilatation was performed. (E) The working channel was moved in the direction of the black arrow, using the inclined baffle to protect the nerve and to enlarge the operable space under the operating scope.

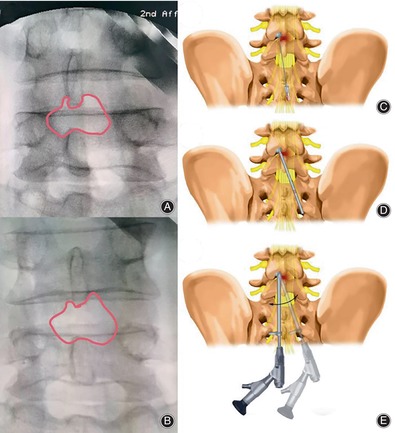

## Introduction

Lumbar disc herniation (LDH) is a common cause of lower back pain, imposing significant economic and health burdens on society and families.^1^ Following the failure of conservative treatment, open or minimally invasive surgery is typically required.^2^ The advancement of minimally invasive surgery technology has led to the increased use of percutaneous endoscopic lumbar discectomy (PELD) in treating LDH. PELD encompasses two techniques: percutaneous endoscopic transforaminal discectomy (PETD)^3^ and percutaneous endoscopic interlaminar discectomy (PEID).^4^

The L4/5 and L5/S1 intervertebral discs are the primary lesion sites in LDH.^5^ Anatomical research indicates that L4/5 intervertebral disc herniation is primarily treated with PETD, whereas L5/S1 herniation is more commonly addressed with PEID.^6^ Research has established that the L4/5 interlaminar space is situated below the L4/5 intervertebral disc level. Furthermore, the smaller size of the L4/5 intervertebral space poses challenges for accessing the disc with PEID spinal endoscopy.^7^ PETD, using the natural anatomical channels, causes less disruption to normal anatomy and significantly reduces long‐term adverse postoperative effects in LDH treatment. Therefore, PETD is the preferred surgical option among most minimally invasive spine surgeons for treating L4/5 disc herniation.^8^ However, in the endoscopic treatment of L5/S1 disc herniation, PEID is often chosen due to factors such as a narrow foramen, obstruction by the high iliac crest, and a large S1 superior articular process.^9^, ^10^, ^11^

Recently, researchers have explored the use of PEID for L4/5 disc herniation, employing techniques such as spinal endoscopy combined with laminoplasty and posterior arthroplasty.^12^ However, these procedures can significantly damage the spinal bone structures and potentially compromise the stability of the lumbar spine.^13^, ^14^ Although it has been reported that the interlaminar approach for L4/5 disc herniation yields postoperative outcomes comparable to PETD, a comprehensive summary and description of the criteria for selecting surgical approaches, methods, and procedural details are lacking.^7^ We have invented a new technology, modified percutaneous endoscopic interlaminar discectomy (MPEID), which can improve short‐term postoperative outcomes and significantly shorten fluoroscopy time. Also, it employs an inclined baffle to gently reposition the spinal cord and nerve root, enhancing the protection of these structures. The purposes of this study were: (i) to evaluate the efficacy of MPEID through postoperative follow‐up and imaging data, and (ii) to evaluate the safety of the MPEID procedure.

## Methods

### General Information

A retrospective analysis was conducted on patients with L4/5 LDH at the Second Affiliated Hospital of Nanchang University from June 2020 to June 2021. The studies involving participants were approved by the Ethics Committee of The Second Affiliated Hospital of Nanchang University (Review [2020] No. [086]). Informed consent was obtained from the patients for the surgical procedure.

Inclusion criteria included: (i) precise unilateral L4/5 disc herniation diagnosis; (ii) presentation of unilateral radicular symptoms, with or without lower back pain; (iii) MRI confirmation of unilateral herniation consistent with symptomatic side; (iv) CT imaging showing no calcification in the herniated lumbar disc. (v) Ineffectiveness of conservative treatment over 3 months; and (vi) surgery intervention (MPEID or PETD). Exclusion criteria included: (i) any history of lumbar spine surgery; (ii) presentation of bilateral radicular symptoms; (iii) MRI confirmation of unilateral herniation inconsistent with symptomatic side; (iv) LDH combined with conditions such as endplate osteochondritis, lumbar spondylolisthesis, lumbar spine tumor, and other related diseases; and (v) incomplete medical records.

Seventeen patient underwent MPEID, and there were 97 cases of PETD that met the inclusion criteria. To match the sample size of the MPEID group, we selected 17 patients through the random number table method. The baseline characteristics of the patients are detailed in Table 1. Effectiveness was evaluated using the visual analogue scale (VAS),^15^ Oswestry disability index (ODI),^16^ and modified MacNab criteria.^17^ The MPEID group included nine males and eight females, with an average age of 38.12 ± 10.57 years, a preoperative ODI score of 51.35 ± 10.65, a VAS score for back pain of 5.41 ± 2.18, and a VAS score for leg pain of 6.53 ± 1.66. The PETD group comprised eight males and nine females, with an average age of 33.29 ± 11.82 years, a preoperative ODI score of 48.00 ± 11.62, a VAS‐back score of 4.94 ± 1.98, and a VAS‐leg score of 7.12 ± 1.73. No statistically significant differences were found in the preoperative data between the two groups.

**TABLE 1:** Patients characteristics.

	PETD	MPEID	*p*‐value
Patient (male/female)	17 (8/9)	17 (9/8)	*p =* 0.73 (χ^2^ = 0.12)
Age (years)	33.29 ± 11.82	38.12 ± 10.57	*p =* 0.22 (T value = 0.51)
Affected side (left/right)	11/6	8/9	*p =* 0.49 (χ^2^ = 0.48)
Fluoroscopy occurrence(times)	9.71 ± 1.05	6.47 ± 1.00	*p <* 0.001 (T value = 0.70)
Operative time (minutes)	91.65 ± 14.04	65.41 ± 12.61	*p <* 0.001 (T value = 0.83)
VAS‐Back (Pre‐operation)	4.94 ± 1.98	5.41 ± 2.18	*p =* 0.52 (T value = 0.38)
VAS‐Back (12 months after operation)	2.06 ± 1.25	1.76 ± 1.09	*p =* 0.47 (T value = 0.42)
VAS‐Leg (Pre‐operation)	7.12 ± 1.73	6.53 ± 1.66	*p =* 0.32 (T value = 0.60)
VAS‐Leg (12 months after operation)	1.12 ± 0.60	0.82 ± 0.64	*p =* 0.18 (T value = 0.25)
ODI (Pre‐operation)	48.00 ± 11.62	51.35 ± 10.65	*p =* 0.39 (T value = 0.26)
ODI (12 months after operation)	12.24 ± 2.56	11.71 ± 2.91	*p =* 0.58 (T value = 0.61)
MacNab (12 months after operation, E/G)	12/5	15/2	*p = 0.23* (*χ* ^*2*^ = 1.47)

### Surgical Indications

The transforaminal approach was preferred in cases with: (i) LDH with a narrow lamina space of < 20 mm; and (ii) lateral and extreme lateral LDH. The interlaminar approach was chosen for: (i) LDH obstructed by a high iliac crest or lumbar transverse process; (ii) giant type, extrusion type, and sequestered LDH; and (iii) LDH revealing an interlaminar space of 20 mm or more.^18^

### Surgical Procedures

#### Surgery Steps for PETD Group

**FIGURE 1:**
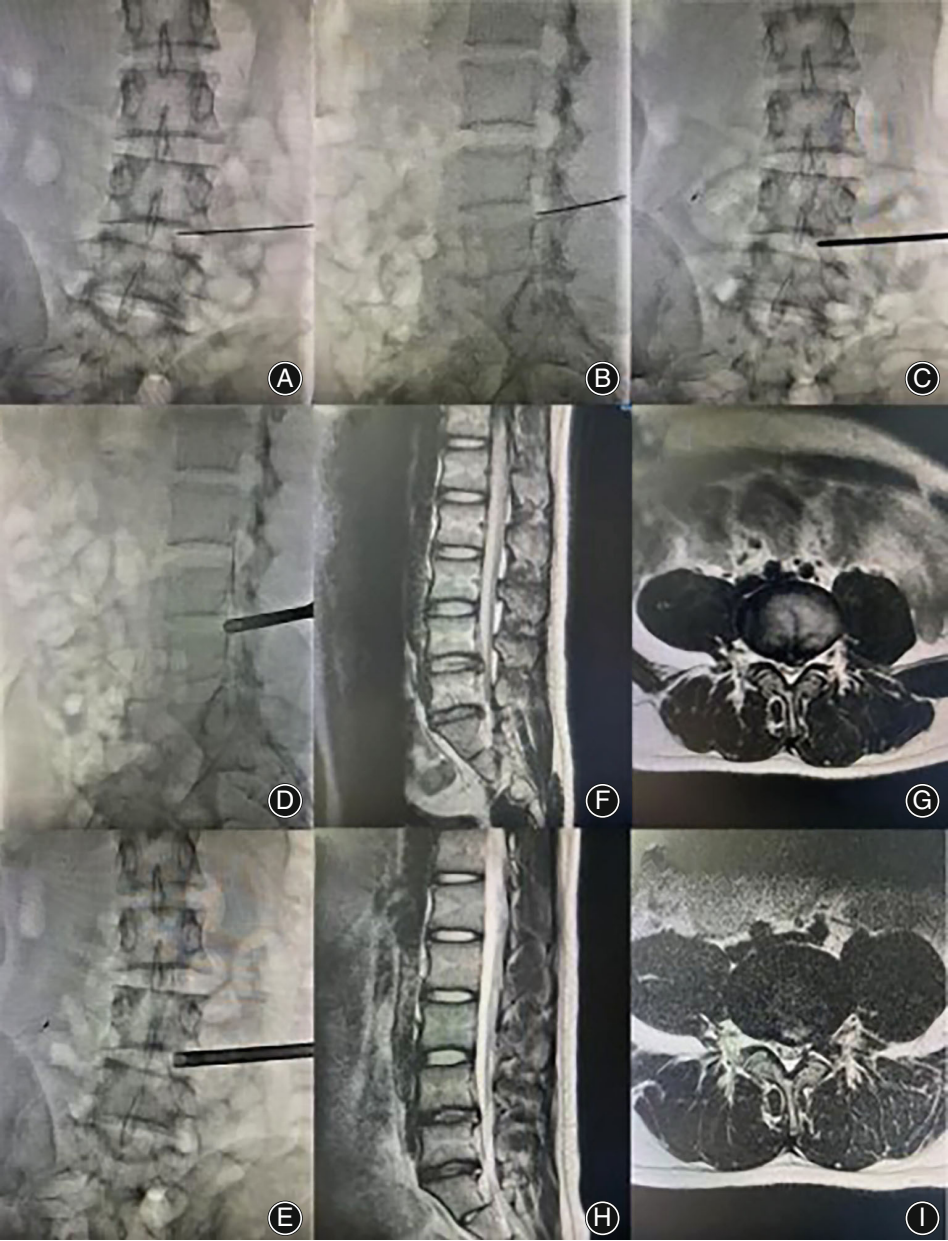
PETD intraoperative fluoroscopy and preoperative and postoperative. MRI (A–E) Intraoperative radiographs. (F and G) Preoperative MRI, H and I: MRI 3 months after surgery.

1. Positioning and preoperative preparation: patients can be positioned in either lateral decubitus or prone position. In this study, patients were positioned in a lateral decubitus position on a radiolucent table with C‐arm fluoroscopy assistance. The procedure involved marking, disinfection, draping, and local infiltration anesthesia using 20 mL of 1% lidocaine and 20 mL of 0.9% saline.
2. Anesthesia and intervertebral discography: under fluoroscopic guidance, an 18G spinal needle was inserted into the ventral side of the superior articular process at L5 for local anesthesia (Figure 1AB). The needle was carefully advanced into the target intervertebral disc to inject a mixture of methylene blue and iohexol (1:9) for discography, which was confirmed by fluoroscopy.
3. Soft tissue dilation: a guidewire was inserted through the needle, followed by needle removal. An 8 mm incision was made using a sharp knife, and sequential tract dilatation was carried out (Figure 1C). If necessary, an 8.5 mm inner diameter trephine can be used to remove the lateral bone of the L5 superior articular process and expand the intervertebral foramen.
4. Placement of working channel: the working cannula was positioned, and its placement was verified by fluoroscopy (Figure 1D,E). The endoscope, light source, and radiofrequency electric knife were connected. Intervertebral disc decompression was performed to create space for nerve root exposure.
5. Removal of protruded intervertebral disc and decompression of nerve roots: the traversing nerve roots were exposed by removing the ligamentum flavum, followed by decompression of the ventral nerve roots. Intervertebral disc calcifications, if present, can be removed using a microscopic grinding drill. If necessary, the exiting nerve roots were examined postoperatively using an endoscope to observe blood vessel filling and pulsation on the nerve root surface.
6. Surgical completion and wound suture: the procedure was considered complete if the nerve root was relaxed and no active bleeding was present. The incision was closed with 3–0 absorbable sutures.

### Surgery Steps for MPEID Group

**FIGURE 2:**
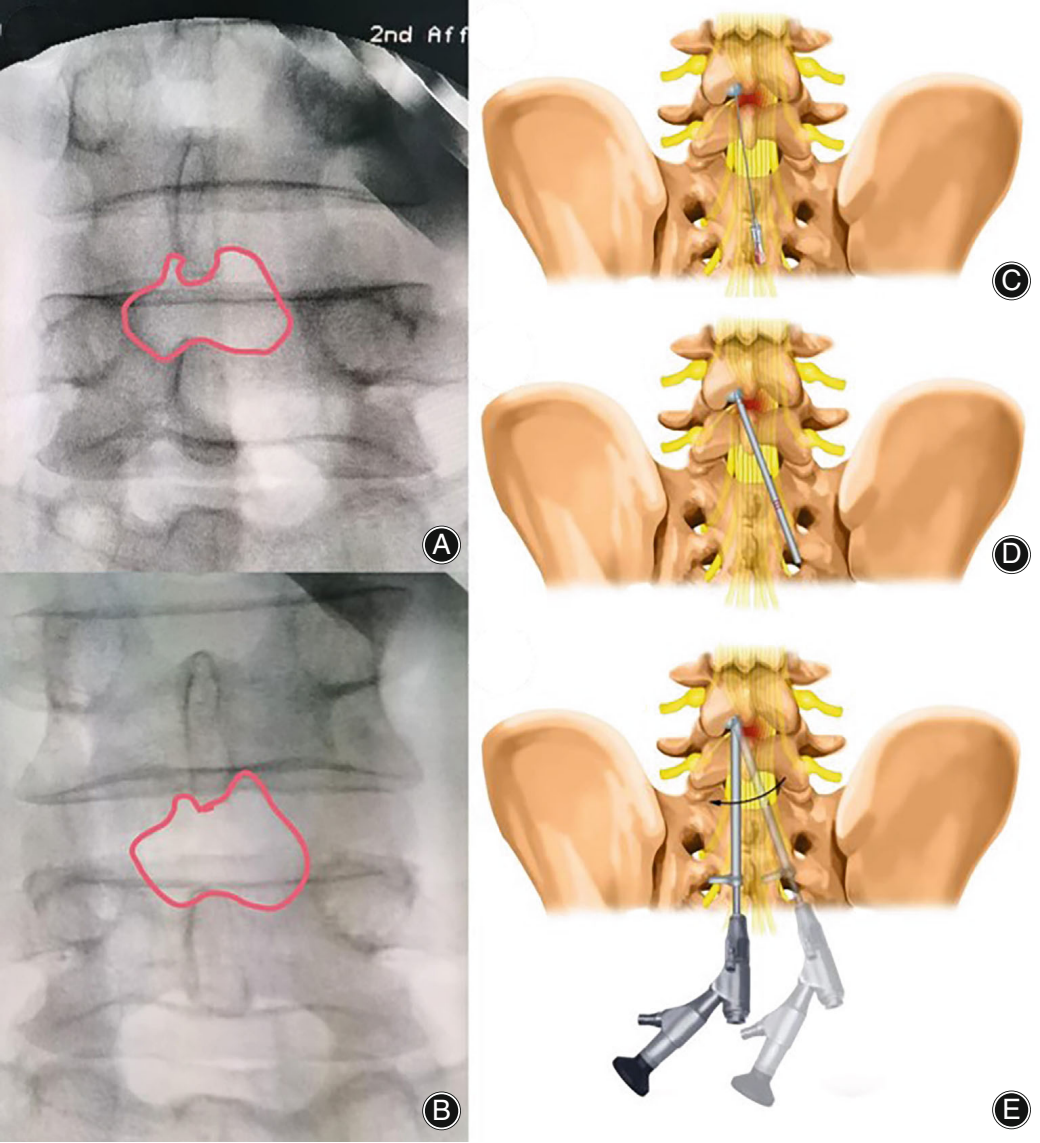
Schematic diagram of the MPEID spinal endoscopy technique for L4/5 disc herniation. (A and B) Interlaminar space with and without a postural cushion on X‐ray. (C) An 18‐G needle was inserted from the lower end of the L5 spinous process to the most lateral edge of the L4 lamina on the affected side. (D) The sequential tract dilatation was performed. (E) The working channel was moved in the direction of the black arrow, using the inclined baffle to protect the nerve and to enlarge the operable space under the operating scope.

**FIGURE 3:**
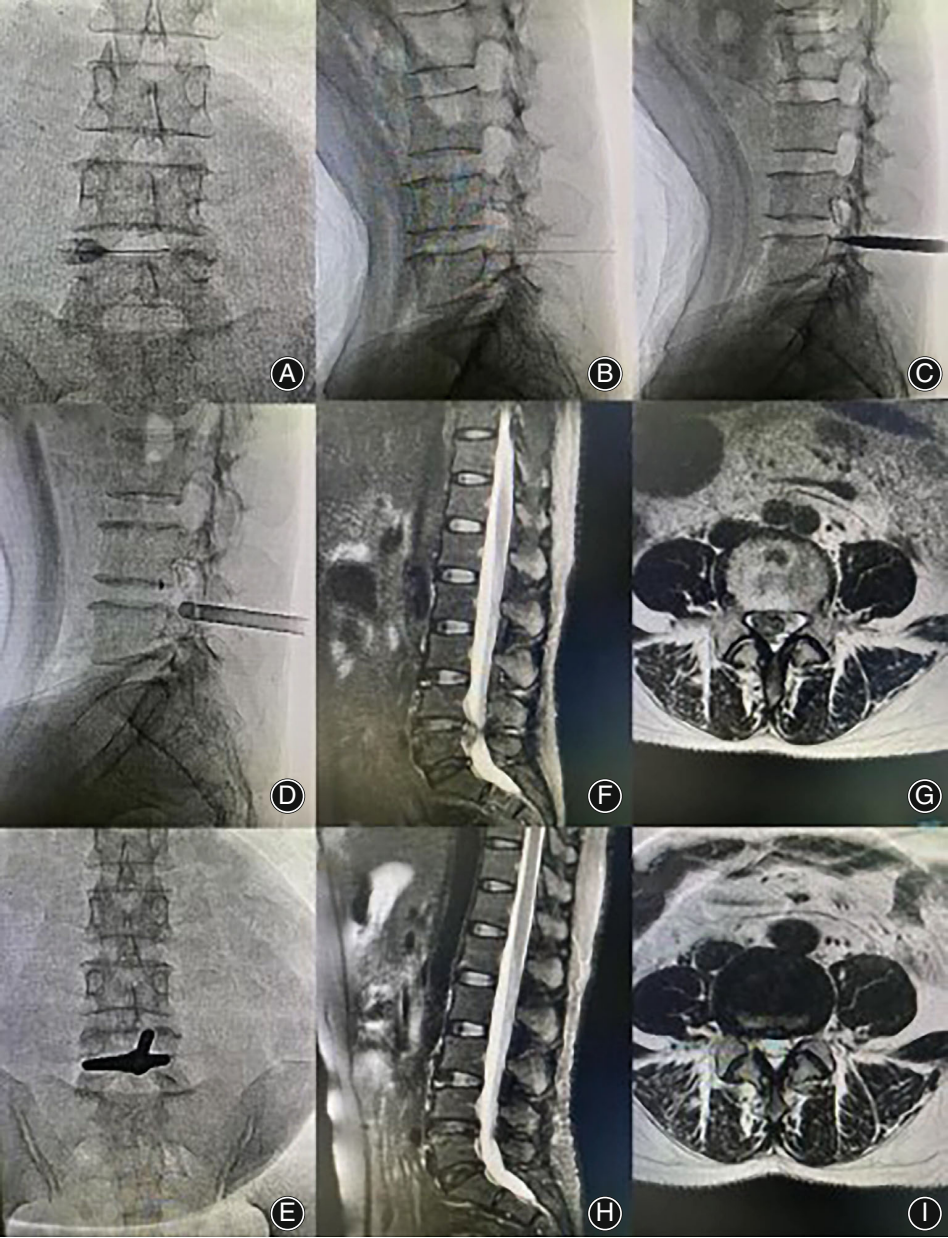
MPEID intraoperative fluoroscopy and preoperative and postoperative MRI. (A–E) Intraoperative radiographs, (F and G) Preoperative MRI, H and I: MRI 3 months after surgery.

1. Positioning and preoperative preparation: patients were positioned prone with nursing pads under the abdomen and underwent fluoroscopy (Figure 2A,B and S1). The procedure began with marking, disinfection, and draping, followed by local anesthesia using 20 mL of 1% lidocaine and 20 mL of 0.9% saline.
2. Anesthesia and intervertebral discography: with fluoroscopic assistance, an 18‐G needle was inserted from the upper end of the L5 spinous process to the most lateral edge of the L4 lamina on the affected side (Figures 2C, 3A,B). After layer‐by‐layer infiltration anesthesia, the needle was obliquely inserted to penetrate the ligamentum flavum and into the disc, followed by injection of methylene blue and iohexol (1:9) for discography. Fluoroscopy was used to confirm injection into the disc.
3. Soft tissue dilation: a guidewire was introduced through the spinal needle, which was then removed. An 8 mm incision was created using a sharp knife, and sequential tract dilatation was performed (Figures 2D and 3C).
4. Placement of working channel: the working cannula was positioned and verified using fluoroscopy (Figure 3D,E). The endoscope, light source, and radiofrequency electric knife were connected. The working cannula and endoscope were adjusted upright slowly, and the cannula was rotated gradually. The inclined baffle at the front of the cannula was used to gently move the nerve root and spinal cord to the healthy side for further protection (Figure 2E).
5. Removal of protruded intervertebral disc and decompression of nerve roots: the herniated intervertebral disc was accurately targeted, and direct decompression of the herniated disc and nucleus pulposus was performed. Postoperative endoscopic observation of the nerve root surface was conducted to check for blood vessel filling and pulsation.
6. Surgical completion and wound suture: the operation was completed if the nerve root was relaxed and no active bleeding was observed. The incision was closed with 3–0 absorbable sutures.

### Postoperative Management

Dexamethasone (5 mg, once daily) and mannitol (125 mL, twice daily) were administered intravenously on the day of surgery and the following day. Postoperatively, the surgical patients are typically discharged within 1–5 days, and all of these patients are required to wear a waist support brace when sitting, standing, or walking for 3 weeks. None of the patients were allowed to lift heavy objects during the first 3 months. Early functional rehabilitation exercises were also crucial, and each patient was instructed to perform five sets of 20 bilateral straight leg raises daily.

### Postoperative Evaluation Indicators

The number of intraoperative fluoroscopy occurrences in our study were recorded using the C‐arm machine's built‐in tracking system. Surgery time, number of intraoperative fluoroscopy occurrences, the ODI, VAS, and modified MacNab scores were compared between the two groups at 1 day, 3 months, and 12 months post‐surgery.

### Statistical Methods

Data analysis was conducted using IBM SPSS software (version 26.0, Armonk, NY, US). A Shapiro–Wilk test was utilized to assess the normality of data distribution. Statistical comparisons between the two groups were conducted using a *t*‐test for continuous variables and a chi‐square test for categorical variables to analyze the demographic data and outcome measures. The preoperative and postoperative outcome measures were compared using a paired *t*‐test. A *p*‐value < 0.05 was considered to indicate statistical significance. *p < 0.05; **p < 0.01; ***p < 0.001.

## Results

### Intraoperative Procedures

**FIGURE 4:**
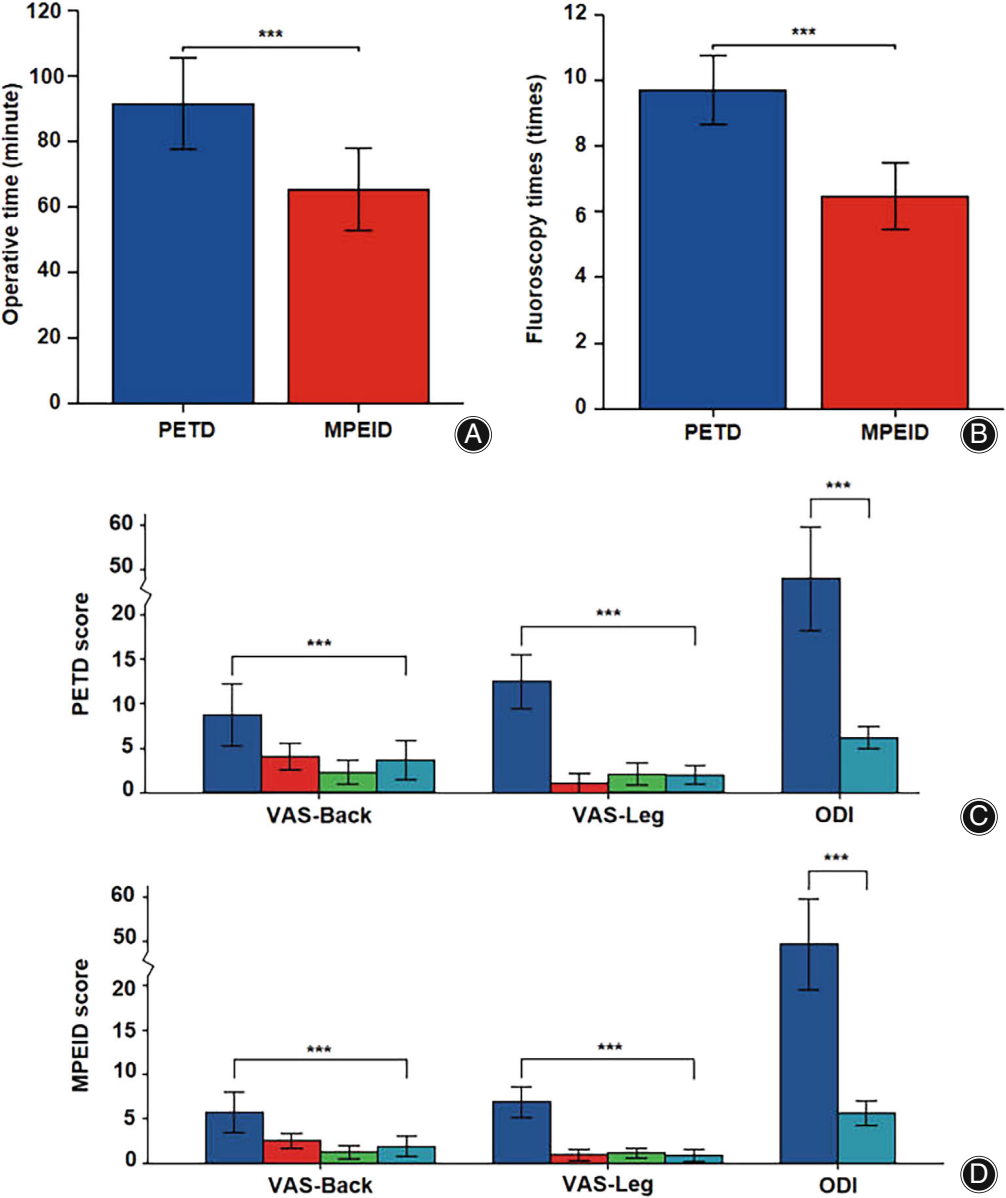
Clinical efficacy evaluation. (A) Compared to PETD group, the MPEID group showed a significant decrease in operative time. (B) Compared to PETD group, the MPEID group showed a significant decrease in fluoroscopy times. (C) In the PETD group, there was a significant decrease in VAS‐back, VAS‐leg, and ODI scores at postoperative day 1, 3 months, and 12 months. (D) In the MPEID group, there was a significant decrease in VAS‐back, VAS‐leg, and ODI scores at postoperative day 1, 3 months, and 12 months. *p < 0.05; **p < 0.01; ***p < 0.001.

All surgeries were completed, with significant postoperative pain relief reported in patients from both surgical groups. The average operative time in the PETD group was 91.65 ± 14.04 min, and the average operative time in the MPEID group was 65.41 ± 12.61 min (*p* < 0.001). In the PETD group, the fluoroscopy occurrences averaged 9.71 ± 1.05 times, with fluoroscopy occurrences averaging 6.47 ± 1.00 (*p* < 0.001) in the MPEID group (Table 1, Figure 4A,B).

### Evaluation of the Clinical Effect

All 34 patients were followed for at least 12 months (Table 1, Figure 4C,D). In the MPEID group, the preoperative VAS‐back score of 5.41 ± 2.18 decreased to 1.76 ± 1.09 (*p* < 0.001) postoperatively, while in the PETD group, it dropped from 4.94 ± 1.98 to 2.06 ± 1.25 (*p* < 0.001). The VAS‐leg score in the MPEID group declined from 6.53 ± 1.66 preoperatively to 0.82 ± 0.64 (*p* < 0.001) postoperatively and in the PETD group, from 7.12 ± 1.73 to 1.12 ± 0.60 (*p* < 0.001). Similarly, the ODI score in the MPEID group reduced from 51.35 ± 10.65 to 11.71 ± 2.91 (*p* < 0.001), and in the PETD group, from 48.00 ± 11.62 to 12.24 ± 2.56 (*p* < 0.001). According to the Modified MacNab evaluation criteria, all 17 patients in the MPEID group achieved either excellent (15 patients) or good (two patients) postoperative results, and in the PETD group, 12 patients achieved excellent and five good results (*p =* 0.23). Overall, both groups showed favorable outcomes without any reported adverse outcomes.

### Complications

No serious complications, such as nerve root injury, dural tear, postoperative infection, or hematoma, were observed in either group following surgery. In the MPEID group, one patient experienced slight numbness, which resolved within a day.

## Discussion

This study introduces a novel technique, MPEID, for treating L4/5 LDH and evaluates its clinical effectiveness and safety. MPEID, specifically, offers advantages such as reduced operation time and decreased frequency of fluoroscopy usage. The retrospective clinical study demonstrated improvements in postoperative VAS‐back, VAS‐leg, ODI, and modified MacNab evaluations among the 34 patients who underwent MPEID and PETD for L4/5 disc herniation.

### Clinical Efficacy of MPEID

Similar to previous studies, clinical outcomes all improved significantly using two surgical methods.^19^, ^20^, ^21^ However, using the MPEID, the fluoroscopy usage and operative time were significantly reduced compared to the conventional foraminal approach. Wang *et al*. demonstrated the average operative time in the PETD was 80.4 ± 18.0 min and the fluoroscopy occurrences averaged 17.1 ± 8.7 times,^22^ and the average operative time in the MPEID group was 65.41 ± 12.61 min, with fluoroscopy occurrences averaging 6.47 ± 1.00 times. The fluoroscopy usage and operative time were both significantly decreased in the MPEID group.

The working cannula directly broke through the ligamentum flavum, enhancing procedural efficiency. Also, the posterior puncture distance in MPEID is shorter, and the procedure is simpler compared to the transforaminal approach. This method allows direct penetration through the ligamentum flavum following targeted puncture of herniated intervertebral discs, notably shortening operative time relative to the conventional Ruetten interlaminar approach for spinal endoscopic nucleus pulposus discectomy.^2^ Moreover, MPEID aligns more closely with spine surgeons' routine practices in terms of patient positioning and anatomical approach, facilitating easier adoption and promotion of this endoscopic spinal treatment for L4/5 disc herniation. No serious complications were observed in either group following surgery. In the MPEID group, one patient experienced slight numbness, which resolved within a day. Previous studies have shown that, the incidence of numbness varied from 0 to 17.88%.^23^, ^24^ So, only one patient experienced numbness in this study was found to be acceptable.

### Advantages of MPEID

**FIGURE 5:**
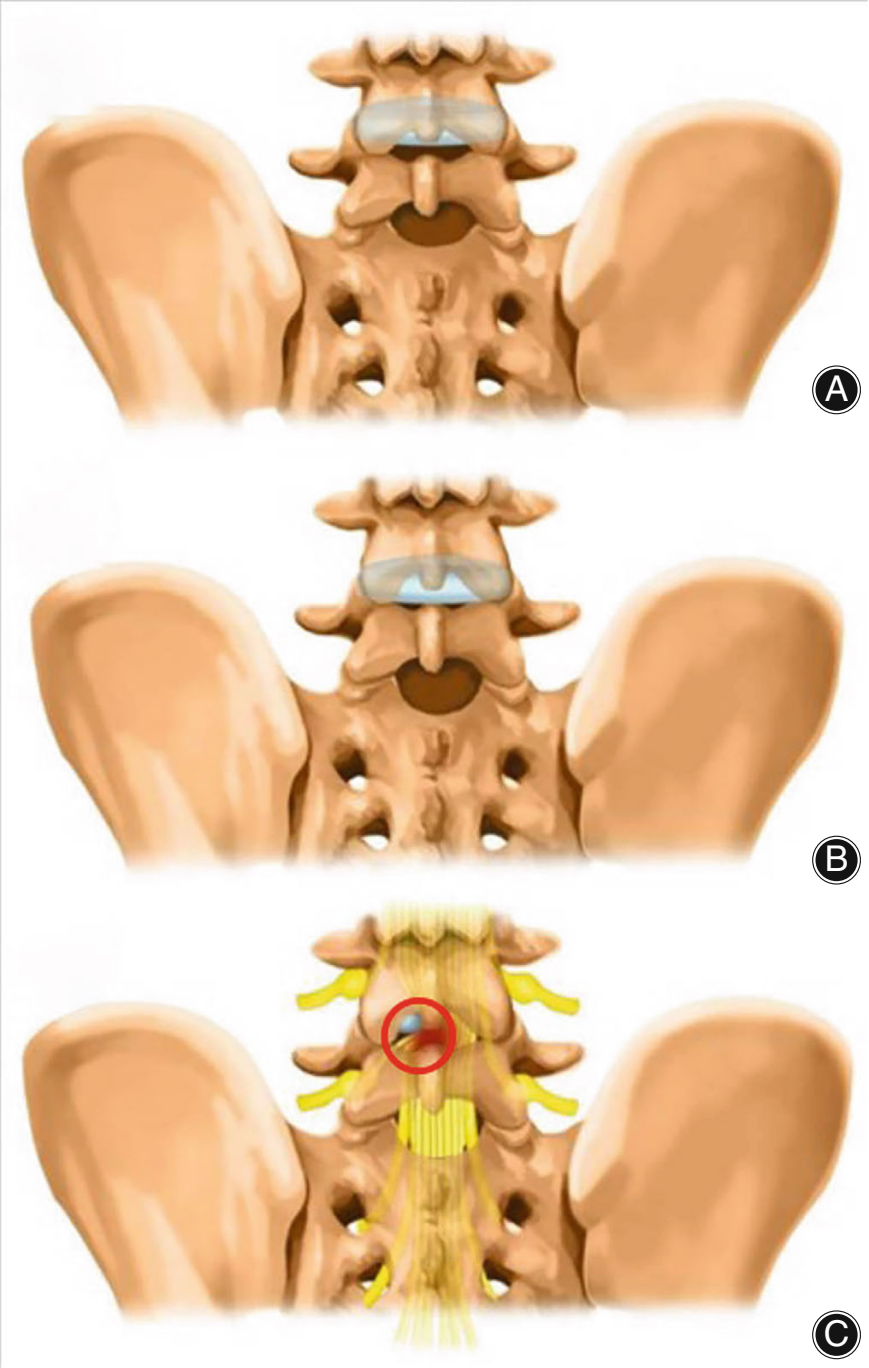
Diagram of the safety of the procedure. (A and B) Interlaminar space of L4/5 laminae interval space was widen and the angle between the L4/5 plate gap and the intervertebral disc was reduced. (C) A safe zone was created within the intervertebral space with the herniated disc's displacement of the nerve root.

The proposed MPEID is a safer, more accurate, and convenient spinal endoscopic technique. The narrow interlaminar space of L4/5 laminae interval space and the higher level of the L4/5 disc compared to the L4/5 laminae interval space caused a challenge for L4/5 PEID.^14^, ^25^ Positioning with postural nursing pads can significantly widen this space and reduce the angle between the L4/5 plate gap and the intervertebral disc, facilitating the placement of the surgical working cannula (Figure 5A,B). Also, MPEID is less optimal for central disc herniations, it is particularly advantageous for lateral herniations, especially those causing unilateral spinal cord compression. Lumbar MRI provides valuable information on the orientation of nerves and spinal cord and their spatial relationship with other intervertebral discs.^26^ The herniated disc's displacement of the nerve root creates a safe zone within the intervertebral space (Figure 5C). An oblique puncture from the outer lower edge of the L4 vertebra aligns with human anatomy, effectively avoiding spinal nerves and enhancing puncture safety.^12^, ^15^ Furthermore, the working cannula's upright placement through gradual rotation, coupled with an inclined baffle at the front, enables the repositioning of the nerve root and spinal cord towards the healthier side, effectively protecting these structures.^16^

The results demonstrate favorable short‐term postoperative outcomes with this technique, as confirmed by postoperative imaging showing complete removal of the nucleus pulposus and effective nerve decompression. The objective of LDH surgery is to alleviate nerve root compression while minimizing surgical trauma.^27^ The MPEID is conducted using a posterior spinal endoscope, aligning with spine surgeons' familiar practices and offering a relatively short learning curve.^28^ Compared to endoscopic spinal surgery *via* the intervertebral foraminal approach, MPEID is more efficient and less time‐consuming, preserving bone structure and lumbar spine stability. Additionally, it employs an inclined baffle to gently reposition the nerve root and spinal cord, enhancing the protection of these structures. Overall, MPEID is a promising technique for wider adoption.

In contrast to PETD for L4/5 disc herniation, which often fails to target the herniated disc nucleus pulposus directly, resulting in little improvement on the postoperative MRI,^17^ MPEID allows for a relatively direct targeting and safe decompression. This study found that MPEID for L4/5 disc herniation yielded good imaging outcomes, as evidenced by preoperative and postoperative lumbar MRI comparisons. If as is hoped, that this technique can be applied, patients will experience less surgery time with a safer procedure.

### Limitations

This study has several limitations. First, the participant pool was limited to a single center, resulting in a relatively small sample size. Second, the follow‐up period was brief. Further multi‐center, prospective studies involving a larger cohort of patients and extended follow‐up durations are necessary to validate the findings and enhance their generalizability.

## Conclusion

In conclusion, both PETD and MPEID yield satisfactory outcomes in treating L4/5 LDH. MPEID, in particular, has advantages in terms of shorter operation time and fewer fluoroscopy occurrences. For L4/5 disc herniations with an interlaminar space of < 2 cm and MRI scans that do not indicate a surgical safe zone, PETD is the recommended treatment approach. Conversely, MPEID is more suitable for L4/5 disc herniations where the interlaminar space exceeds 2 cm, and MRI scans reveal a surgical safe zone. In cases with specific surgical indications, MPEID can serve as a viable alternative to PETD for L4/5 disc herniation treatment.

## Author contributions

JY and XC contributed to the study concept and design. PX and XM participated in the data acquisition and analysis. TW performed the surgery and perioperative management on the patient. PX, DH and JY wrote the manuscript with contributions from all co‐authors. All authors contributed to the article and approved the submitted version.

## Funding Information

This study was supported by the National Science Founding of China (No. 81860397), the Natural Science Foundation of Jiangxi Province of China (No. 20202ACBL206012), and the project of the Science and Technology Department of Jiangxi Province (No. 20202BCD42018).

## Conflict of Interest Statement

The authors have no competing interests.

## Ethics Statement

This study was performed at Department of Orthopaedics, The Second Affiliated Hospital of Nanchang University. And this study was approved by the Ethics Committee of The Second Affiliated Hospital of Nanchang University (Review (2020) No. (086)). All the procedures are conducted in accordance with the guidelines and regulations.

## Supporting information


**Figure S1.** Patient position during surgery.

## References

[1] Scaturro D , Asaro C , Lauricella L , Tomasello S , Varrassi G , Letizia MG . Combination of rehabilitative therapy with Ultramicronized Palmitoylethanolamide for chronic low Back pain: an observational study. Pain Ther. 2020;9(1):319–326. 31863365 10.1007/s40122-019-00140-9 PMC7203351

[2] Yu H , Zhu B , Liu X . Comparison of percutaneous endoscopic lumbar discectomy and open lumbar discectomy in the treatment of adolescent lumbar disc herniation: a retrospective analysis. World Neurosurg. 2021;151:e911–e917. 33989822 10.1016/j.wneu.2021.05.007

[3] Yeung AT , Tsou PM . Posterolateral endoscopic excision for lumbar disc herniation: surgical technique, outcome, and complications in 307 consecutive cases. Spine. 2002;27:722–731. 11923665 10.1097/00007632-200204010-00009

[4] Ruetten S , Komp M , Godolias G . A new full‐endoscopic technique for the interlaminar operation of lumbar disc herniations using 6‐mm endoscopes: prospective 2‐year results of 331 patients. Minim Invasive Neurosurg. 2006;49:80–87. 16708336 10.1055/s-2006-932172

[5] Kapetanakis S , Gkantsinikoudis N , Charitoudis G . Implementation of percutaneous Transforaminal endoscopic discectomy in competitive elite athletes with lumbar disc herniation: original study and review of the literature. Am J Sports Med. 2021;49(12):3234–3241. 34491150 10.1177/03635465211032612

[6] Schultz A , Andersson G , Ortengren R , Haderspeck K , Nachemson A . Loads on the lumbar spine. Validation of a biomechanical analysis by measurements of intradiscal pressures and myoelectric signals. J Bone Joint Surg Am. 1982;64:713–720. 7085696

[7] Huang K , Chen G , Lu S , Lin C , Wu S , Chen B , et al. Early clinical outcomes of percutaneous endoscopic lumbar discectomy for L4‐5 highly Down‐migrated disc herniation: Interlaminar approach versus Transforaminal approach. World Neurosurg. 2021;146:e413–e418. 33353758 10.1016/j.wneu.2020.10.105

[8] Wu H , Hu S , Liu J , He D , Chen Q , Cheng X . Risk factors involved in the early and medium‐term poor outcomes of percutaneous endoscopic Transforaminal discectomy: a single‐center experience. J Pain Res. 2022;15:2927–2938. 36132995 10.2147/JPR.S380946 PMC9484800

[9] Wang D , Xie W , Cao W , He S , Fan G , Zhang H . A cost‐utility analysis of percutaneous endoscopic lumbar discectomy for L5‐S1 lumbar disc herniation: Transforaminal versus Interlaminar. Spine (Phila Pa 1976). 2019;44(8):563–570. 30312274 10.1097/BRS.0000000000002901

[10] Mo X , Shen J , Jiang W , Zhang X , Zhou N , Wang Y , et al. Percutaneous endoscopic lumbar diskectomy for axillar herniation at L5–S1 via the transforaminal approach versus the Interlaminar approach: a prospective clinical trial. World Neurosurg. 2019;125:e508–e514. 30710722 10.1016/j.wneu.2019.01.114

[11] Gao A , Yang H , Zhu L , Hu Z , Lu B , Jin Q , et al. Comparison of Interlaminar and Transforaminal approaches for treatment of L5 /S1 disc herniation by percutaneous endoscopic discectomy. Orthop Surg. 2021;13(1):63–70. 33274579 10.1111/os.12831 PMC7862146

[12] Cheng L , Cai H , Liu Z , Yu Y , Li W , Li Q . Modified full‐endoscopic Interlaminar discectomy via an inferior endplate approach for lumbar disc herniation: retrospective 3‐year results from 321 patients. World Neurosurg. 2020;141:e537–e544. 32492545 10.1016/j.wneu.2020.05.234

[13] Lin H , Zhang S , Wu G , Jin J , Liu L . Two different access intervertebral foraminoscopic techniques for treating L4/5 disc herniation. China Orthopaedic Injury. 2019;32(10):904–909. 10.3969/j.issn.1003-0034.2019.10.006 32512959

[14] Li Y , Wang B , Lv G , Li L , et al. Application of interlaminar gap‐forming technique in treating L4/5 disc herniation by complete endoscopic surgery via interlaminar approach. Chin J Spine Cord. 2017;27(3):193–199.

[15] Huang H , Hu H , Lin X , Wu C , Tan L . Percutaneous endoscopic interlaminar discectomy via inner border of inferior pedicle approach for downmigrated disc herniation: a retrospective study. J Orthop Surg Res. 2022;17(1):359. 35864515 10.1186/s13018-022-03245-8 PMC9306037

[16] Xie TH , Zeng JC , Li ZH , Wang L , Nie HF , Jiang HS , et al. Complications of lumbar disc herniation following full‐endoscopic Interlaminar lumbar discectomy: a large, single‐center. Retrospect Stud Pain Physician. 2017;20(3):E379–E387. 28339437

[17] Heo DH , Lee DK , Lee DC , Kim HS , Park CK . Fully endoscopic Transforaminal lumbar discectomy for upward migration of upper lumbar disc herniation: clinical and radiological outcomes and technical considerations. Brain Sci. 2020;10(6):363. 32532092 10.3390/brainsci10060363 PMC7349390

[18] Tonosu J , Oshima Y , Shiboi R , Hayashi A , Takano Y , Inanami H , et al. Consideration of proper operative route for interlaminar approach for percutaneous endoscopic lumbar discectomy. J Spine Surg. 2016;2(4):281–288. 28097245 10.21037/jss.2016.11.05 PMC5233847

[19] Feng F , Xu Q , Yan F , Xie Y , Deng Z , Hu C , et al. Comparison of 7 surgical interventions for lumbar disc herniation: a network meta‐analysis. Pain Physician. 2017;20(6):E863–E871. 28934804

[20] Ruetten S , Komp M , Merk H , Godolias G . Full‐endoscopic interlaminar and transforaminal lumbar discectomy versus conventional microsurgical technique: a prospective, randomized, controlled study. Spine (Phila Pa 1976). 2008;33(9):931–939. 18427312 10.1097/BRS.0b013e31816c8af7

[21] Sencer A , Yorukoglu AG , Akcakaya MO , Aras Y , Aydoseli A , Boyali O , et al. Fully endoscopic interlaminar and transforaminal lumbar discectomy: short‐term clinical results of 163 surgically treated patients. World Neurosurg. 2014;82(5):884–890. 24907438 10.1016/j.wneu.2014.05.032

[22] Wang Z , Jian F , Wu H , Wang X , Wang K , Duan W , et al. Treatment of upper lumbar disc herniation with a Transforaminal endoscopic technique. Front Surg. 2022;9:893122. 10.3389/fsurg.2022.893122 35574546 PMC9096648

[23] Ahn Y , Lee SH , Park WM , Lee HY , Shin SW , Kang HY . Percutaneous endoscopic lumbar discectomy for recurrent disc herniation: surgical technique, outcome, and prognostic factors of 43 consecutive cases. Spine (Phila Pa 1976). 2004;29(16):E326–E332. 15303041 10.1097/01.brs.0000134591.32462.98

[24] Perez‐Cruet MJ , Fessler RG , Perin NI . Review: complications of minimally invasive spinal surgery. Neurosurgery. 2002;51(5 Suppl):S26–S36. 12234427

[25] Yang J , Yang Q , Tian L , Wang B , Liu Y . Differences in L4/L5 lamina gap morphology in hyperflexed kneeling and prone positions and their significance in percutaneous endoscopic discectomy via interlaminar approach for lumbar disc herniation. J Spine Surg. 2018;16(5):289–292.

[26] Jia J , Ding R , Liu X , Li W , Xiong X , Wu T , et al. Coronal magnetic resonance imaging of three‐dimensional fast‐field echo with water‐selective excitation improves the sensitivity and reliability of identification of extraforaminal lumbar disc herniation. J Int Med Res. 2019;47(12):6053–6060. 31662019 10.1177/0300060519882546 PMC7045647

[27] Dowling TJ , Munakomi S , Dowling TJ . Microdiscectomy. StatPearls [Internet]. Treasure Island (FL): StatPearls Publishing; 2023. 32310444

[28] Wang Y , Wu J , Wang T , Liu Y , Jiang M , Wang Z , et al. Modified lumbar foraminoplasty using a power‐aided reciprocating burr for percutaneous transforaminal endoscopic lumbar discectomy: a technical note and clinical report. Front Surg. 2023;5:1091187. 10.3389/fsurg.2022.1091187 PMC9849748 36684228

