# Supplementary figures and images for: Modified Percutaneous Endoscopic Interlaminar Discectomy through the Near‐spinous Process Approach for L4/5 Disc Herniation: A Retrospective Clinical Study
Source: Orthop Surg. 2024 Mar 31;16(5):1064–72. doi: 10.1111/os.14031 (PMC11062861; doi:10.1111/os.14031)

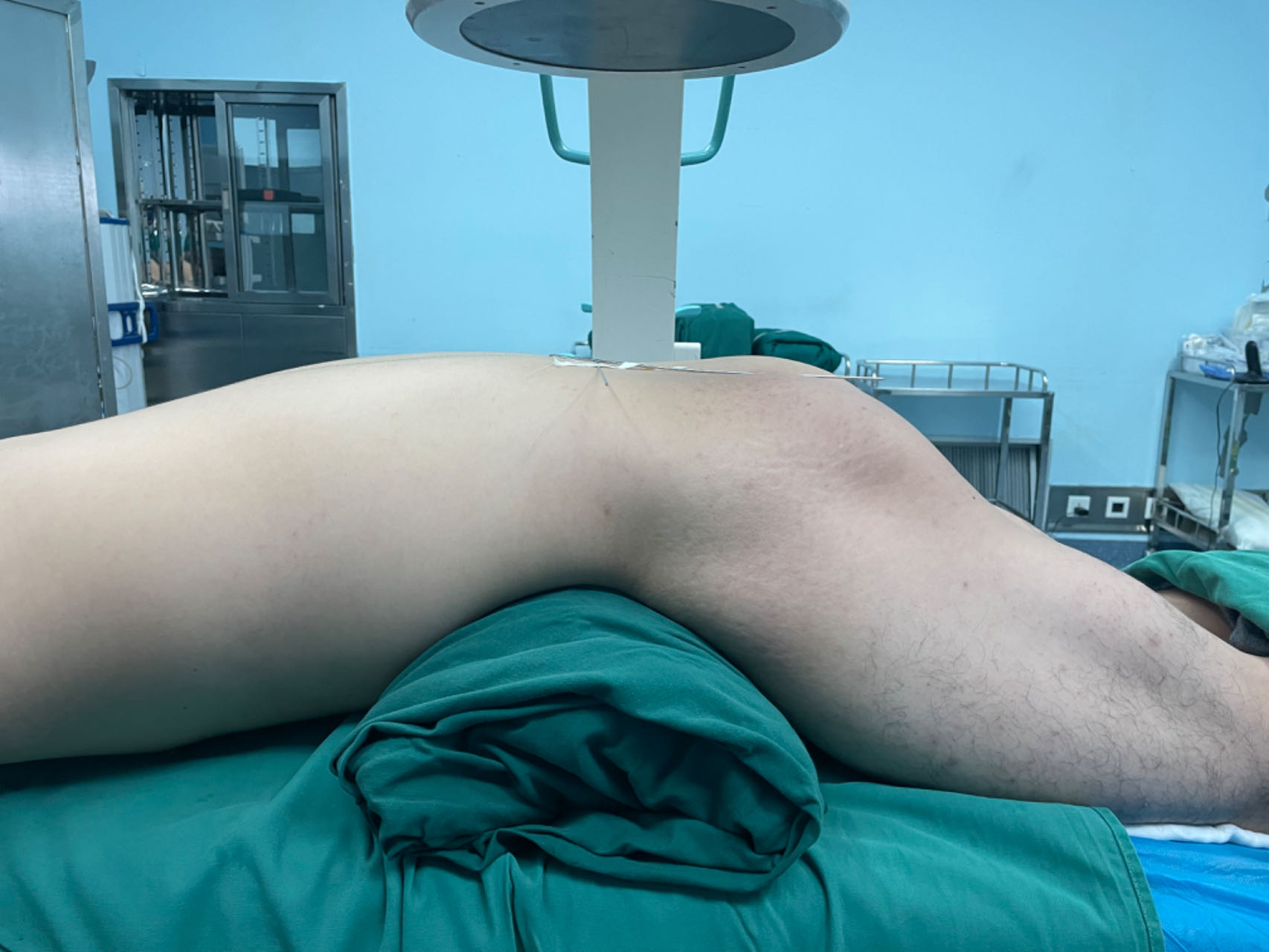

Supplement: Supplementary file 1 — Figure S1. Patient position during surgery. [file OS-16-1064-s001.jpg]
